# Palliative care *stay* room – designing, testing and evaluating a gamified social intervention to enhance palliative care awareness

**DOI:** 10.1186/s12904-023-01166-9

**Published:** 2023-04-20

**Authors:** Carla Reigada, Anna Sandgren, Sonia Rivas, Ana Carvajal, Santiago Hermida-Romero, Edgar Benítez, Guillem Ripoll, Inés Olza, Carlos Centeno, Beatriz Gómez

**Affiliations:** 1 ATLANTES Global Observatory of Palliative Care, Institute for Culture and Society, University of Navarra, Pamplona, Spain; 2 Navarra Institute for Health Research (IdiSNA), Pamplona, Spain; 3 Center for Collaborative Palliative Care, Department of Health and Caring Sciences, Linnaeus University, Växjö, Sweden; 4 School of Education and Psychology, University of Navarra, Pamplona, Spain; 5 School of Nursing, University of Navarra, Pamplona, Spain; 6 School of Economics and Business, University of Navarra, Pamplona, Spain; 7 Emotional Culture and Identity Project, Institute for Culture and Society, University of Navarra, Pamplona, Spain; 8 School of Communication, University of Navarra, Pamplona, Spain

**Keywords:** Palliative care, Stay room, Gamified social intervention, Students, University, Palliative care awareness

## Abstract

**Introduction:**

The message of palliative care can be promoted using creative thinking and gamification. It can be an innovative strategy to promote changes in behaviour, promote thinking, and work on skills such as empathy.

**Aim:**

Design, test and evaluate a gamified social intervention to enhance palliative care awareness among young university students from non-health background.

**Methods:**

Participatory action research study with mixed methods, Design Thinking and using the Public Engagement strategy. Forty-three undergraduate students participated in a Palliative Care *Stay* Room and completed the Test of Cognitive and Affective Empathy (TECA) before and after the game. At the end of the game, a ten-minute debriefing was held with the participants, which was concluded with an open conversation. The content analysis was done independently and the sum of the scores of each dimension was compared before and after the activity.

**Findings:**

The Stay Room improved the participants’ knowledge and new perspectives about palliative care. Before the game, their views focused on the end of life and after the game on their values, highlighting the dedication of the healthcare professionals who do not treat death but the life until death. After de game, participants (N = 43: female = 23; male = 20; x̄ 19.6 years old) presented higher values in perspective adoption (intellectual ability to put oneself in the other’s place) p = 0.046 and in emotional understanding (ability to recognize emotional states) p = 0.018, and had high scores on empathic joy (p = 0.08).

**Conclusion:**

Gamification can be used in teaching and transmitting positive attitudes. Palliative Care and can help young university students to think positively about care issues.

**Supplementary Information:**

The online version contains supplementary material available at 10.1186/s12904-023-01166-9.

## Introduction

The promotion of positive attitudes towards end-of-life issues has been a concern in raising awareness of palliative care in society [1–3]. Palliative Care issues should be part of the socio-political debate [4]. As it is a human right, all citizens should have access to this service and be properly informed of its real meaning and objectives [5, 6]. Palliative care focuses on attending to human suffering, derived from a situation of serious, advanced illness [7]. By having awareness, information and knowledge, people are more willing to listen and talk about this topic and are not afraid to address, among other issues, their concerns about life and death [4, 8].

To achieve this, educational activities can promote positive attitudes, and schools and universities are privileged places to stimulate the humanity of the young towards the seriously ill [9–11]. But how can this be done? On the one hand, the increase of palliative care seminars and courses in medical and nursing schools is evident, yet the challenge is to take this knowledge of humanized care outside the science faculties and so spread the message of palliative care as widely as possible [4, 12]. On the other hand, how does one teach compassion or humanity? Clearly not through exams or the usual teaching activities. It is necessary to think out of the box to find creative pedagogical solutions, while constantly measuring their feasibility and impact [11, 13].

In recent years, several studies have been conducted for game-based education of patients, families, and health professionals, highlighting its cognitive, emotional, and social benefits. However, there are clear gaps in the use and gamification in palliative and end-of-life care [14]. A recent scoping review on games in palliative care showed that serious games have been used to raise awareness and educate different groups in society since 1993 [15]. Simulations and role-plays are more commonly used in training future health professionals, while card games are more commonly used to talk about advanced care planning issues. Those games are used to train aspects related to communication, but very few record the gains before and after the games [15]. For example, the mission ‘Bed Race, The End of Life Game’ is an educational board game for teaching palliative and end of life care, which was tested with 251 medical students. The game proved to be feasible and effective because it increased the participants’ knowledge. In addition, students highlighted that playing as a team increased the positive experience, they had with palliative care issues [14]. It was also found in another study that the “End-of-Life Conversation Game” increased the confidence of chaplains-in-training to have end-of-Life conversations with people in palliative care [16].

Games can be a way to promote the expression of emotions, to engage, and to increase creativity and motivation. In education, they can trigger positive attitudes and small changes in behaviour, thinking, and work on soft skills (e.g., empathy and social intelligence). Educational games (e.g., empathy games) should be better explored by palliative care professionals to work with society on the compassion model [16, 17]. Empathy is a multidimensional construct composed of cognitive, affective, and behavioural dimensions. Some studies point out that women register higher levels of empathy and, specifically, have a greater sensitivity and knowledge of issues related to palliative care [18, 19]. Davis Harris [14] describes empathy as the set of constructs that include the processes of putting oneself in the other’s place and affective and non-affective responses; it is the general capacity to resonate with others emotional states irrespective of their valence - positive or negative [20]. We believe that empathy is the key that can enable people to develop compassionate behaviour. As Sinclair argues, empathy can contribute to the interpersonal construction involving awareness and intuition, the cognitive aspect, and the affective aspect [17]. Although complex, it is through empathy that learning happens, as it allows us to work on attention (i.e., being focused on the situation); retention (having the ability to remember a situation); reproduction (practising what we have seen and felt); and motivation (responding to the impulse that moves us). These characteristics are described by Bandura as the steps of social learning [21]. We assume in this study that the game can improve empathy towards palliative care, and this may be a key strategy to increase their awareness of the topic. Recognising that there is a lack of research studies on empathy enhancing awareness of palliative care, and after the conclusion of our last study where students stated that they wanted to know more about palliative care in a positive way [4], the aim of this study is to design, test and evaluate a gamified social intervention to enhance palliative care awareness.

## Methods

### Research design

A mixed-methods approach was used during the three-phase study, combining both quantitative and qualitative techniques and instruments. Phase 1 is characterized by the designing of a Palliative Care Stay Room (Intervention), Phase 2 corresponds to its testing, and Phase 3 its evaluation. Participatory Action Research (PAR) using the Design Thinking (DT) approach and adopting the Public Engagement in Responsible Research and Innovation (PE-RRI) strategy was the methodology adopted for this study.

### Justification of research methodology

PAR is comprehended as progressive research, consisting of cyclical steps: (i) planning, (ii) action, (iii) observation, and (iv) results, involving the participants since the beginning. It is considered the preferred methodology in educational science that, in a practical way, develops and tests measurable solutions to a specific problem and context. We choose this method as it is focused on generating change, promoting knowledge and applicable results, and reflecting systematically on the action and the problem studied [22]. In addition, we used Design Thinking methodology as it works on human-centred problem solving, enhancing creativity and innovation of final products (solutions/interventions). This methodological procedure also uses cyclical steps described as: (i) empathy, (ii) definition, (iii) ideation, (iv) prototyping, and (v) testing. In education, Design Thinking was first applied in design schools where students from different faculties and disciplines integrated interdisciplinary projects, working together to find disruptive solutions [23]. As part of the PE-RRI strategy we involved the subjects of the study in the research team, as involvement increases the quality and validation of the research findings and promotes the enhancement of knowledge between researchers and beneficiaries. The PE-RRI strategy encourages the co-production of knowledge jointly by researchers and society actors during the all-research process [24]. Two students (taking degrees in nursing and education) were integrated from the beginning in research design, together with five researchers (four experts in Palliative Care and one statistician), three university professors (from education, communication, and public administration), and one expert in Design Thinking.

### Participants

For the phase 1 of this study (Palliative Care Stay Room Design), researchers, university professors and students at the University of Navarra were invited by email to design the palliative care stay room. These people were from different areas, and they were already involved in the EnPositive-PAL project, in which they had already collaborated on two studies: one focused on the perception of university students of palliative care [4] and the other, which aimed to find an innovative strategy among the university community to promote palliative care [2].

For the phase 2 and 3 (Palliative Care Stay Room Test and Evaluation), the inclusion criteria to participate in the study were: undergraduate students at University of Navarra taking clinical and non-clinical degrees, up to 23 years old, able to understand the Spanish language. The exclusion criteria included students over 24 years of age, and all those who were attending another university. The students at the University of Navarra were invited by social media and by the university newsletter to sign up for a gamified experience called Stay Room, advertised as follows: “Dare to stay in the room –Come and play on caring for vulnerable people and their environment with a maximum duration of 60 min”.

### Study context

This study was carried out at the University of Navarra in Spain. The University of Navarra is a Christian-inspired non-profit institution with teaching, research, and healthcare resources. The University has seven different campuses in Spain, 16 schools, 38 undergraduate degrees with about 8,705 undergraduate students from 109 nationalities. The University of Navarra 2021–2025 strategy adopted sustainable development, care for people (where we can find Palliative Medicine), and the environment as the keystones of the university projects.

### Procedures

#### Phase 1 – designing the palliative care stay room

Six people designed the Palliative Care Stay Room [four experts in Palliative Care (CR), Communication (BG), Design Thinking (SH), Pedagogy (SR) and two students taking Nursing (DL) and Pedagogy (BV) degrees]. Described as similar to an Escape Room – a game, with a specific physical space, in which players discover clues, solve puzzles and carry out tasks inside a room, with the aim of escaping as quickly as possible to move on to the next phase – the aim of our game was not to escape from the room but to challenge players to stay to experience, explore and resolve enigmas together with the game master (who leads the game – a young patient). The design was co-created during six work sessions made during one month. Literature on gamification, communication and palliative care, clinical case studies, brainstorming, brainwriting, visual thinking and storytelling were resources used as a methodological basis to design the Palliative Care Stay Room. The Phase 1 design was built in three steps: (1) Theme and storyline, (2) Game ground-rules, and (3) Practical issues. Table I summarizes which aspects were discussed in each step, and the supplement material of this paper includes the full script of the game. The Palliative Care Stay Room (the intervention) was designed to have four “rooms” or “spaces” with their own decor, easy to build with materials and allowing plenty of flexibility in creating other materials for the whole game. This intervention was designed with sustainability in mind. Accessing the detailed description in the supplementary material of this article, it is possible to quickly set up and taken down the game. In this supplement you will find the instructions and all the necessary materials. The videos, cards, paintings, etc., produced in this Stay Room are in Spanish language and can be requested directly to the correspondence author of the study.

**Table 1:** Phase 1 Structure to Design a Palliative Care Stay Room

Steps	Topics discussed
1. Theme and storyline	- Defining a clear goal: what experience must participants go through to incite empathy and acquire knowledge? - Choosing a story (using storytelling technique to write the beginning, the body, and the end of the story) - Imagining the scenario (using storyboarding technique to visualize details) - How many rooms do we have in our history? (assuming that a Room represents a moment of progress in the story) - Selecting one specific aim for each Room and linking it to the final message
2. Game ground-rules	- Playing duration - How many players per game (minimum and maximum) - Rules for players during the game - What and how players will gain versus lose - Selecting two or three enigmas to solve per Room
3. Practical Issues	- Create a list of objects we need in each Room - Where we can get and buy it (budget) - Which materials do we need to create or select so that the gaming experience can be as dynamic as possible (e.g., videos, sounds, music, light effects) - Consider selecting spaces that feature electricity and technology - Contact some volunteers to help with logistic issues (instructions, assembly, disassembly, maintenance, observation…) - Consult different people from different fields (companies that create escape rooms, audio-visual experts, interior designers, actors, gamers, other researchers with experience in gamification, etc.) - Involve civic and political stakeholders - Select ten people from your target group to run the pre-test

#### Phase 2 – testing the palliative care stay room

A week before the start of the Palliative Care Stay Room, four students were invited to test the game to detect any flaws. Their feedback was essential to improving the logistics and procedures and to facilitating harmony between the game and the learning experience itself. On the day and time scheduled, each group of students went to the Medical Simulation Centre of University of Navarra and were received by a researcher who ensured that the consent form was properly filled out. A brief explanation of the game was given in Room 1 (Reception) without mentioning that it was an experience related to Palliative Care. In total, there were five prepared Rooms, and the aim was that undergraduate students could find messages and solve enigmas based on the story of Ricardo, a fictitious patient and the master of the game, who audio-guided them through the circuit. The goal was to meet Ricardo’s needs and fill his life with life (#*LlenadeVidaTuVida*, the hashtag used for the game).

#### Phase 3 – evaluating the palliative care stay room

When students registered themselves through a link or QR code, they automatically received the following information: “1. Welcome! Gather a group of four University of Navarra students and assign a group name. 2. Select a day and time to play. 3. Agree to fill out the Cognitive and Affective Empathy Test [25] on empathy before and after the game. 4. Share your experience. 5. This game is free of charge and the participants will receive an Amazon voucher worth €10”. The participants were also informed that they needed to contribute their impressions of the learning experience in a videotaped debriefing at the end of the game.

**Fig. 1:**
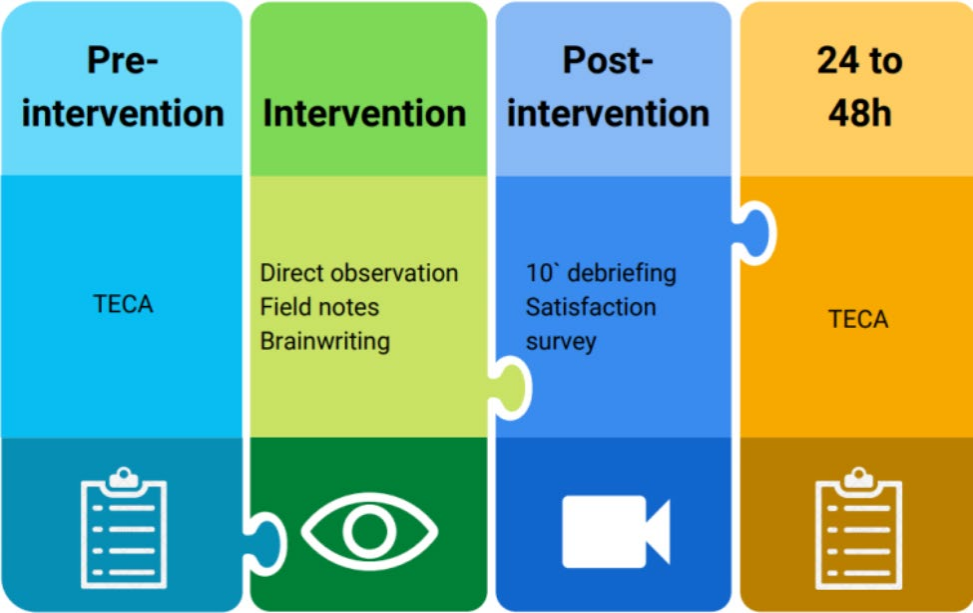
Evaluation measures

The registration on the Google form recorded the consent of the participants. Before starting the Stay Room, students filled out the Cognitive and Affective Empathy Test – Spanish version (TECA: Test de Empatía Cognitiva y Afectiva), a global measure of empathy containing 33 items [25]. This self-report measure presents a four-factor structure: (i) Perspective Adoption (PA), referring to the intellectual or imaginative capacity to step into somebody else’s shoes (8 items); (ii) Emotional Understanding (EU), related to the capacity to recognize and understand the emotional states, intentions and impressions of others (nine items); (iii) Empathic Stress (ES) or the ability to share other the negative emotions of others (eight items); and (iv) Empathic Joy (EJ), which refers to the ability to share the positive emotions of others. The same scale was sent to the participants by email 48 h after the game ended. The researchers registered the field notes using direct observation, seeing the interaction of the participants during the game through surveillance cameras and one-way mirrors. In the last Room the participants were invited to leave their reflections in writing. A meeting to share the experience (brief videotape debriefing) was also promoted in each group of participants, and a short online satisfaction survey was given to participants to ascertain the acceptability of the Stay Room, identifying potentials and areas for improvement (Fig. 1).

### Analysis

#### Quantitative data analysis

In a preliminary way, the reliability of the factor measurements was evaluated by calculating TECA Cronbach statistics, where Alpha values of 0.6 for PA, 0.7 for EU, 0.7 for ES, and 0.7 for EJ were obtained. In general, the skewness and kurtosis values had absolute values lower than the two units [25], except for three items of the EJ dimension (2, 4 and 19) and for one item of PA. Given this problem of normality of the data, the scores of the dimensions were calculated by estimating a SEM model through the robust two-stage method [26], before and after the intervention. For the subsequent analyses, the difference between the scores of the different dimensions of empathy after the intervention minus the previous values was taken as the response variable to the calculation; this difference was called *gain*. Initially, the gain was evaluated for the factors of age and sex through uncorrected t-tests. This variable was then evaluated under an analysis of covariance model, in which the gain was presented as a function of the scores of each of the respective scores before the intervention and the factors of sex and age group. For the age group, the respondents were classified into two groups: younger than 20 years, and equal to or older than 20 years. The covariance model was evaluated for the normal distribution of residuals using the Shapiro-Wilk test. The software used was SAS 9.4 [27].

#### Qualitative data analysis

The Palliative Care Stay Room was analysed using the materials produced in the game, the experience of the participants, direct observation, and field notes. In Design Thinking, the analysis of the results happens systematically [23]. All materials resulting from the action (e.g., post-its, drawings, diagrams) were organised into idea blocks and discussed directly within the research group. This phase of direct analysis and synthesis aimed, in a constant and participatory way, to analyse what was being felt in each group of participants. Subsequently, an affinity analysis of the materials (a predictive analysis technique that aims to build correlations between data and interpret them based on their occurrence among participants), was carried out. From here, preliminary categories and themes were found. The transcriptions of seven participant group debriefings (n = 24 students), field notes of the research group and direct observation records were inductively and independently analysed by three independent experts in PC, qualitative analysis, and ethnography (AC, AS, CR). A descriptive analysis was made based on three questions: (1) Has the stay room promoted empathy in students? (2) What were the main take home participants’ messages? (3) How did participants evaluate the game? Constant conversations and discussions on the findings were made to reach a final agreement. The final analysis was discussed with other people of the research group (BG, SH, SR, GR) experts in public management, narrative journalism, linguistic, design thinking and medicine, noting differences, and with one student of pedagogy (B), part of the research team since the beginning of the study.

## Results

**Fig. 2:**
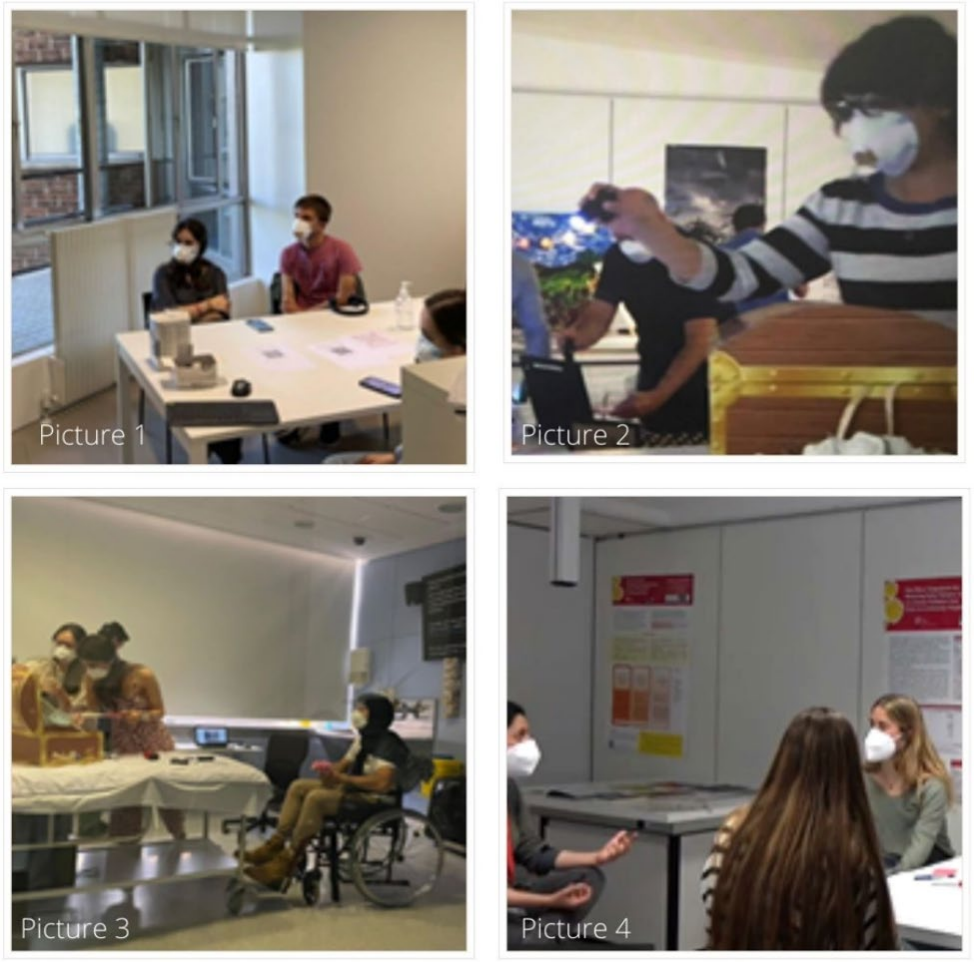
**Palliative Care Stay Room pictures** (Picture 1: Participants listening to Ricardo’s game instructions; Picture 2: Finding the box with values of Richard’s life; Picture 3: Opening Ricardo’s values box; Picture 4: Final debriefing)

The Palliative Care Stay Room was held at the University’s Medical Simulation Centre from May 26th to 28th, 2021. Over three days, forty-three University of Navarra students, divided into ten groups of four and one group of three participants, played the Palliative Care Stay Room for average durations of 60 min per game (see Fig. 2). The average age of students was 19.6 (range 18–23). Twenty-three female and 20 male students with no experience in palliative care field participated (n = 43); most of them were from the first year of university (n = 29). Students from Business, Economics and Governance disciplines (n = 12), Communication field (= 11), Pharmacy and Nutrition (n = 9), Pedagogy (n = 3), Law (n = 2), Philology and philosophy (n = 2), Engineering (n = 1), Architecture (n = 1), Psychology (n = 1), and Nursing (n = 1), participated in the game.

### The palliative care stay room promotes empathy towards palliative care

Initially, the mean values of the scores before and after the intervention were calculated (see Table 2). Where there is an increase in the scores of the “adoption of perspective” and “emphatic joy” scales, and a decrease in the scores of “Emphatic understanding” and “Empathic Stress”.

**Table 2:** Means for all dimensions evaluated before and after the activity

Dimension	Mean of scores (Std. dev.)	
	Before	After
Adoption of Perspective	-0.053 (0.905)	0.079 (0.838)
Emphatic understanding	0.008 (0.857)	-0.012 (0.967)
Emphatic Joy	-0.113 (0.910)	0.169 (0.828)
Empathic Stress	0.043 (0.924)	-0.065 (0.847)

In the uncorrected t-tests (see Table 3), in general, sex seems to be unrelated to the gain of the evaluated dimensions. This observation stands in contrast to age group, which shows that for Adopting Perspective and Emotional Understanding TECA dimensions, older students have higher values in these dimensions.

**Table 3:** Uncorrected t-test for the gain variables associated with each empathic dimension

Variable	Dimension	Levels	Mean	Pr > |t|
Age group	Empathic joy	Older	0.3209	0.345
		Younger	0.1485	
	Adoption of perspective	Older	0.5602	*0.037
		Younger	-0.0069	
	Emotional understanding	Older	0.4541	*0.011
		Younger	-0.1549	
	Empathic stress	Older	0.2213	0.116
		Younger	-0.0904	
Sex	Empathic joy	Female	0.3181	0.118
		Male	0.0203	
	Adoption of perspective	Female	0.2507	0.881
		Male	0.2055	
	Emotional understanding	Female	0.0639	0.625
		Male	0.1952	
	Empathic stress	Female	0.0405	0.973
		Male	0.0329	

* p < 0.05

**Fig. 3:**
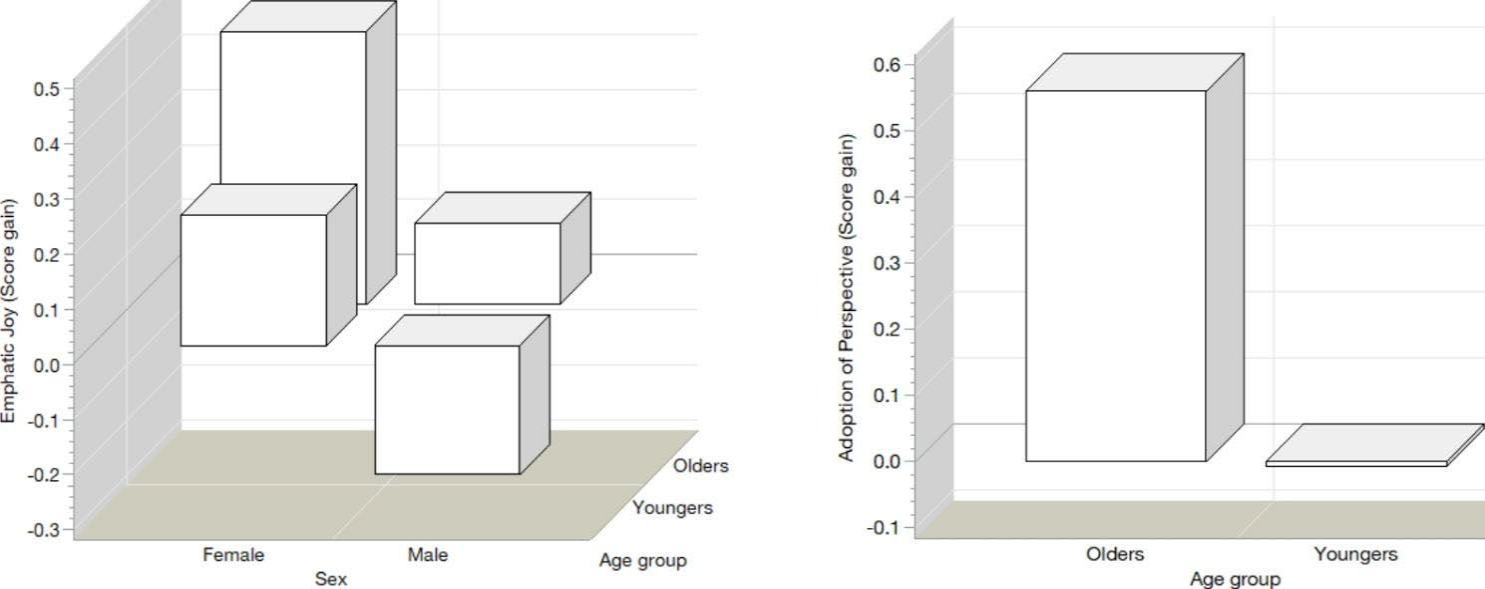
PA gain for the age group factor, corrected for sex and initial values of the PA score

**Fig. 4:**
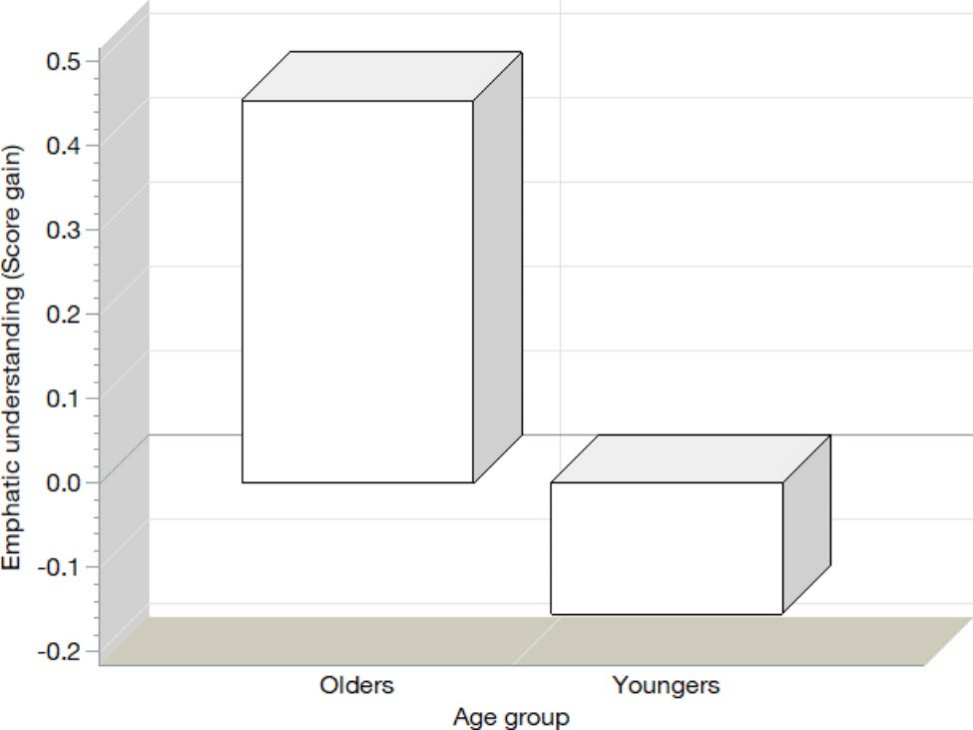
Gain in the US for the age group factor, corrected for sex and initial values of the US score

In the analysis of covariance (see Table 4), where the response of each dimension of empathy is corrected for the factors of sex and age group and, for the initial scores of their respective dimension, the effect of the age group on the dimensions of Adoption of Perspective, Empathic Understanding and Empathic Joy are confirmed (see Fig. 3). A significant effect of sex appears on the Empathic Joy dimension and reveals that older women obtain the greatest gain in this dimension and young men the lowest; some even have negative values, which imply a reduction in their Empathic Joy levels (see Fig. 4).

**Table 4:** Analysis of covariance of the gain responses of each empathic dimension as a function of the initial values of each dimension, the age group, and the sex of the respondent

Gain response	Predictor	Value F	Pr > F
Adoption of perspective	Initial score AP	2.91	0.101
	Age group	4.41	*0.046
	Sex	0.83	0.371
Emotional understanding	Initial score EU	0.89	0.355
	Age group	6.49	*0.018
	Sex	0.31	0.583
Empathic stress	Initial score ES	2.36	0.138
	Age group	1.82	0.189
	Sex	0.35	0.559
Empathic joy	Initial score EJ	4.44	*0.046
	Age group	2.78	0.108
	Sex	8.43	*0.008

* p < 0.05

### Evaluation of student’s empathy before and after the palliative care stay room

#### Providing knowledge

The students reflected on what they understood about palliative care before and after the game. The fact that they did not know the game was about palliative care at the beginning of the game awakened feelings of surprise and shock at the end: ‘There is no such thing as nothing to do with patients! This has a lot of challenges.’ (Student 2). “It even arouses interest in knowing more about it. I think we should spend a day… to see it first-hand. What kind of activities do they (PC team) do? What do they give, talks? What do they do with patients?” (Student 10).

#### New views on the life at the end of life/opening minds

A lot of dedication is needed to work in palliative care, which is not about death, but about living until death. This information must be given; “palliative care seeks to fight for life” (Student 11). “I take with me more than anything a positive message that you must always make the most of life, because you never know what can happen to you” (Student 40). – The students leave the game with the vision to value life, to love and to be with their loved ones. One must take the opportunity to live right now, in this moment, because life can change anytime. At the same time, the students seem more aware that illness can come to you at any age and that Palliative Care brings hope: “You can also get a very tough illness like this one and that, within the negative, which is obvious; there are also ways to get through it” (Student 27).

#### Increasing awareness about palliative care

Students highlight the community’s need to learn about palliative care, which could be done in different ways. Increasing awareness about Palliative Care is needed. Some ideas have come out of the write-storming at the end of the game: “promoting volunteering”, “using cinema” and “animated short films”, “testimonies”, and “social networks/media”. In summary, the game has promoted positive attitudes and awareness that palliative care is about more than death. Moreover, it has promoted actions to make society aware of palliative care and the vulnerability of these patients. The students identify this experience’s key messages, using post-its, as “support”, “accompaniment”, and “love”. They also associated this experience with “life”, “pain”, “care”, “quality”, “optimism”, “empathy”, “hope” and “peace”.

### Game experience

Eighteen of 43 participants answered the satisfaction survey at the end of the Stay Room. Using a scale of 1 to 5, they rated their experience in the Stay Room at 4.72 (min. 4, max.5). The game itself was rated 4.66 (min.3, max.5). Recommendations about content and organisation were made, and the students requested more information on palliative care and suggest that this type of experience should be implemented in other disciplines and courses in the academic field (Table 5).

**Table 5:** Example of participants’ recommendations

Section	Recommendations
Content	- Give more real examples or showing what palliative care is. - Show the care or what is done in palliative care more closely. - A little more information on the current life of Ricardo. - Specify the tools used in palliative care. - Add some more aspects related to the patient’s family. - The evidence could be better integrated with the story. The escape room seems to have a story, but at times the tests (like in the first room) seem unrelated. I liked the part where you had to interact with the eyes on the computer; it would give more play using this technique. Otherwise, I found it very useful, and the experience was amazing.
Organization/Game	- All participants should experience being in the wheelchair (except one to push it). - Follow up with courses. A day accompanying a person in palliative care. - I think that the part where you take things out of the box and put them around the room needs to be explained better. The symbolism is fine, but I didn’t understand it at the time.

## Discussion

Our study concluded that gamification can be used to teach care-related values by enhancing the emotional empathy of university students. After the Palliative Care Stay Room game experience, older students increased their ability to put themselves in the other person’s shoes (perspective-taking) and female students reported feeling more able to share positive emotions with others. Although studies on the use of gamification to foster empathy in young people are limited, one study concluded that their virtual game (involving playing with a dog) boosted empathy and human attitudes young students in Canada. After playing the game, human attitudes improved significantly, with females scoring higher [19]. Also, a study in the field of palliative care aimed to examine how personal and interpersonal aspects were associated with individual knowledge of and attitudes towards palliative care. The results identified that it was women who had more favourable attitudes towards palliative care [18].

Serious games – games designed to provide a learning experience – can be a strategic resource for palliative care education and awareness. They are described in the literature as a means to study and improve the health of populations in different areas, such as mental health [17, 18]. Since the 1990s, these resources have been used in Palliative Care in education, to work on aspects of interdisciplinary communication and transmission of bad news, as well as to address the issue of advance directives [15].

We believe our study describes the first gamified experience in Palliative Care built on a scientific study [4]. After identifying the perceptions and expectations of university students about Palliative Care, solutions have been co-designed to better understand palliative care in a novel way [4]. Thanks to this multi-methodological study, it was concluded that an Escape Room could be a good tool to convey the message and values of palliative care [4]. The researchers would not have come up with this idea on their own as, in fact, many doubts and much reluctance were expressed about applying gamification to such a serious issue as the end of life. But the students have facilitated a new look at play, which was not playing with Ricardo’s life or in Ricardo’s room but playing at being Ricardo and walking in his shoes for an hour to help them understand important issues.

Video games help to foster empathy and even encourage reflections on death [28, 29]. The Stay Room designed for this study is a collaborative game, in which different members of a social group share and reflect together about a gamified real-world experience. It was not an online game or an individual game because we believed that having a physical experience, where you must see, touch, hear and feel, could have much more impact on the undergraduate students. Students are becoming less and less used to social interaction, and the COVID19 pandemic has encouraged this trend [30]. For this reason, we designed a game that would be more memorable. We cannot forget that empathy is further fostered, as seen in the responses from our study, when they literally embody the character; when players sit in the patient’s wheelchair or get as close as possible to their experience. Of course, it is not easy to resort to this kind of dynamic, which allows moving from storytelling to storydoing through playing the story, but it is very effective [31–33]. To achieve this, it is necessary to work on scriptwriting (the way to create storytelling). This technique allows the audience to adapt the characterization of the character to them, which allows feeling more identified and therefore more empathetic. In addition, it allows to know first-hand the character’s thoughts, details of their life that make them more relatable, as well as to see how they look, to hear their voice or touch their belongings, which makes them more real [33, 34].

The social intervention conducted in this study reminds us of the importance of being attentive to the changes and palliative care needs of a new society [35]. Spreading the word and continuing to fight for the rights of citizens to Palliative Care increasingly involves using integrative and innovative technological and community-based models, which are not yet in place in this field [36]. Creativity, and primarily being able to learn from examples that are not from the context in which Palliative Care is studied, currently takes on a key role in the educational field and is seen as the ingredient that can drive behavioural and social change [36, 37]. Palliative care may therefore see an opportunity here, using the social innovation model to transform ways of getting the message across and making people willing to actively engage with the difficult issues around life and death, illness, and wellbeing [36, 37].

There are a few studies that address how empathy can influence positive views of palliative care. Sinclair describes that empathy appears to be an attribute that does not necessarily require action. It is about knowing how to recognize, understand and feel emotionally attached to a person who is suffering [38, 39]. Perhaps, empathy (connection) is the value that, lying between sympathy (strategy) and compassion (action), should be studied more to raise awareness of Palliative Care in society. From our point of view, it would be interesting to understand whether empathic activities, especially interdisciplinary and creative ones, could be the best strategies to promote social discussion of Palliative Care. It even seems to us that empathic activities could contribute to the training of those professionals in society who have roles as informers, influencers, and decision-makers, such as journalists, administrators, political professionals, and lawyers. Reigada [4] also refer to this in their study, highlighting these unlikely agents as the main focus of educational intervention in palliative care to increase knowledge and awareness and bring the topic into the social debate. Working at the university level is working on the future of a caring society [4]. With our study, the University of Navarra has recognised that the interactive way of engaging and producing knowledge about the importance of care could be offered to our university students. Currently, students from Economics, Architecture, Psychology and Education, and Communication, can take a cross-curricular course called Care and Society, which uses innovative teaching techniques such as case studies using the flipped classroom, gamification, and service learning.

### Limitations

The Palliative Care Stay Room is a rapid prototype born out of ideas from a year of research on student perceptions of palliative care. This study did not have a budget capable of supporting the costs of a specialized company to organize a professional Escape Room, so everything was set up by two researchers (CR, BG) with help of a secretary (EB). The preliminary phase of ideation, writing the script, recording the videos, and preparing the materials took three months, which is how long it took to set up the Stay Room. Anti-Covid measures limited the participation of more students. In fact, it was possible for all 43 students to participate on the condition that we had to ensure that all the materials were cleaned between each group playing the game.

## Conclusion

Engaging students is necessary to implement innovative Palliative Care activities in a university setting. Using collaborative play as a tool to enhance learning, communication, and social discussion around Palliative Care can be a good strategy to promote awareness on this topic. Playfulness makes people more receptive to messages and more attentive to what is going on around them. The Palliative Care Stay Room enhanced empathy and positive attitudes of participants towards palliative care. We believe it can be applied in other universities and in any other countries, adapting its script to what is considered most effective for the target audience. It could also be tested in the general population.

## Electronic supplementary material

Supplementary material

## Data Availability

The data that support the findings of this study are available from the University of Navarra, but restrictions apply to the availability of these data, which were used under license for the current study, and so are not publicly available. Data are however available from the authors upon reasonable request and with permission of the Ethics Committee of the University of Navarra.
