# Supplementary material for: Palliative care stay room – designing, testing and evaluating a gamified social intervention to enhance palliative care awareness
Source: BMC Palliat Care. 2023 Apr 20;22:46. doi: 10.1186/s12904-023-01166-9 (PMC10116670; doi:10.1186/s12904-023-01166-9)
Supplement: Supplementary file 1 — Supplementary material [file 12904_2023_1166_MOESM1_ESM.docx]

Supplementary material

**Script of the Palliative Care Stay Room implemented at the University of Navarra**

**Step 1. Theme and storyline**

The goal of the game was to help players to understand and to empathize with a situation where people are dealing with a serious advanced illness and to invite others to be a voice for palliative care. For this purpose, it was decided to recount the life of a specific alleged patient. The resume of the story was: “Ricardo (fictitious name) is a 25-year-old successful painter who, after starting with muscle pain and other severe symptoms, is no longer able to paint, eat or speak. After several exams, Ricardo was diagnosed with Amyotrophic Lateral Sclerosis (ELA). Today, he is followed by the neurologic team and the palliative care team. He is cared for by his girlfriend Maria (fictitious name). Together, they want to pass on a message of life and invite others to be a voice for palliative care”. A character of that age was chosen because it is an age close to the participants and he was given an identity, gestures, and way of speaking that the team discussed with the actor in charge of bringing him to life. Ricardo’s personal Instagram account was created to provide a chronology of his life and because the story is presented as a challenge: “Accompany me through the stages of my life to understand me and to let you know why palliative care is fundamental for me and why I want you to spread the message that it is key in society”. Ricardo supposedly answers the participants’ questions through direct messages, in case they get stuck in any of the tests.

Five different Rooms represent five stages in Ricardo’s life: In the **First Room (Reception),** Ricardo receives the players through a video recorded with his mobile phone inside a car, and tells them about his successes, presents his fiancée Maria and shows the best of his enthusiasm for life (Picture 1). He invites the players to follow his Instagram account (Picture 2), as during the game he will send them messages to allow them to interact in real time. Ricardo offers the players some ultraviolet light lanterns and invites them to come to his studio (Video 1, Picture 1).


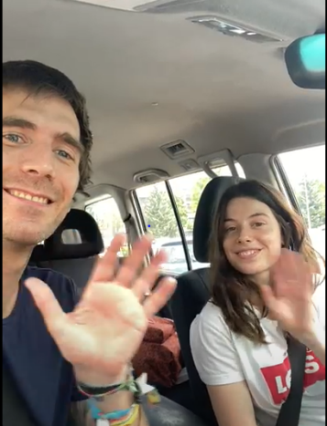

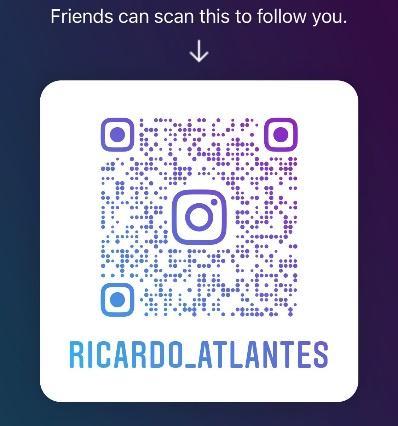


Picture 1

Picture 2

(Picture 1 – Ricardo - master game and fictitious patient) and Maria (Ricardo’s fictitious fiancée); Picture 2 – Ricardo’s Instagram)

The **Second Room (Revelation)** represents Ricardo’s painting studio. Here, the players found many personal objects such as colours, paintings, brushes, books, music... There was a handwritten message in the middle of the Room, inviting them to find a keyword using the lanterns (Picture 3). The letters of the keyword were painted with invisible ink on some objects: the canvas of painting had the letter C, the ping-pong racket the letter U, the ping-pong ball the letter I, a slipper the letter D, a tree of life the letter A, and the safe box the letter R. Players needed to find and write on the board the keyword CUIDAR (meaning CARE) to hear the next video (video 2) of Ricardo. This video marks the turning point of the game, as it is here that Ricardo tells them about his diagnosis (Picture 4). This video also advised players they needed to take the safe box with them during the game. The box is closed with a padlock and represents the most important values of Ricardo’s life.

**
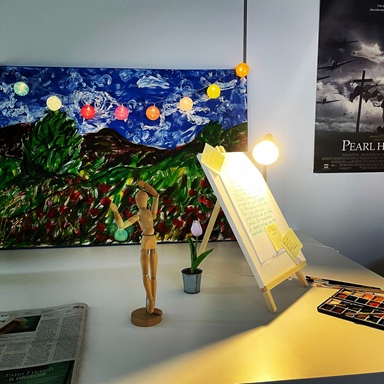

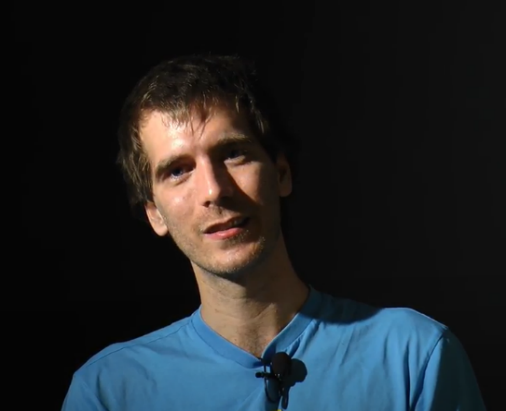
**

Picture 4

Picture 3

(Picture 3– Ricardo’s Studio, Picture 4 – Ricardo (fictitious patient) tells them about his diagnosis)

**Third Room (Waiting Room)**: Before entering the fourth Room, the players get an idea of what Ricardo’s next destination was as they pass through a hospital waiting room. The players saw five messages written by Ricardo, from his diary, which he has left on the chairs each time he has gone to the hospital (Picture 5). They needed to read, understand and organise the messages (Image 5) in correct order to enter the next Room. Each message represents the grief stages described by Küebler Ross (Picture 6): 1. Denial, 2. Anger, 3. Bargaining, 4. Sadness, 5. Acceptance. There was a picture on the waiting room wall with this information.

**
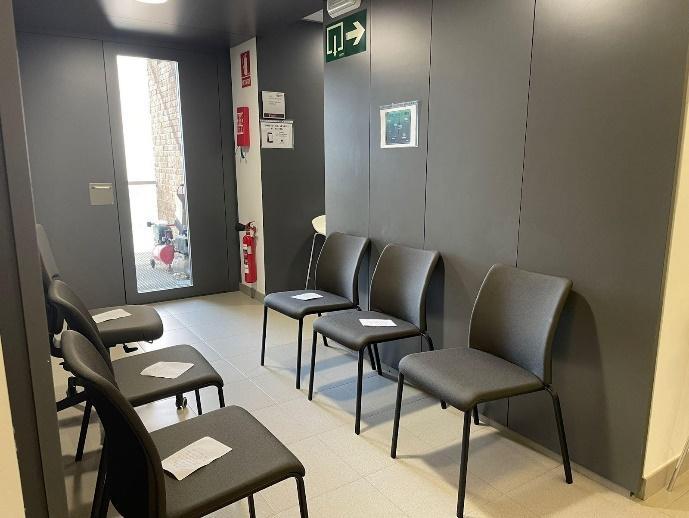

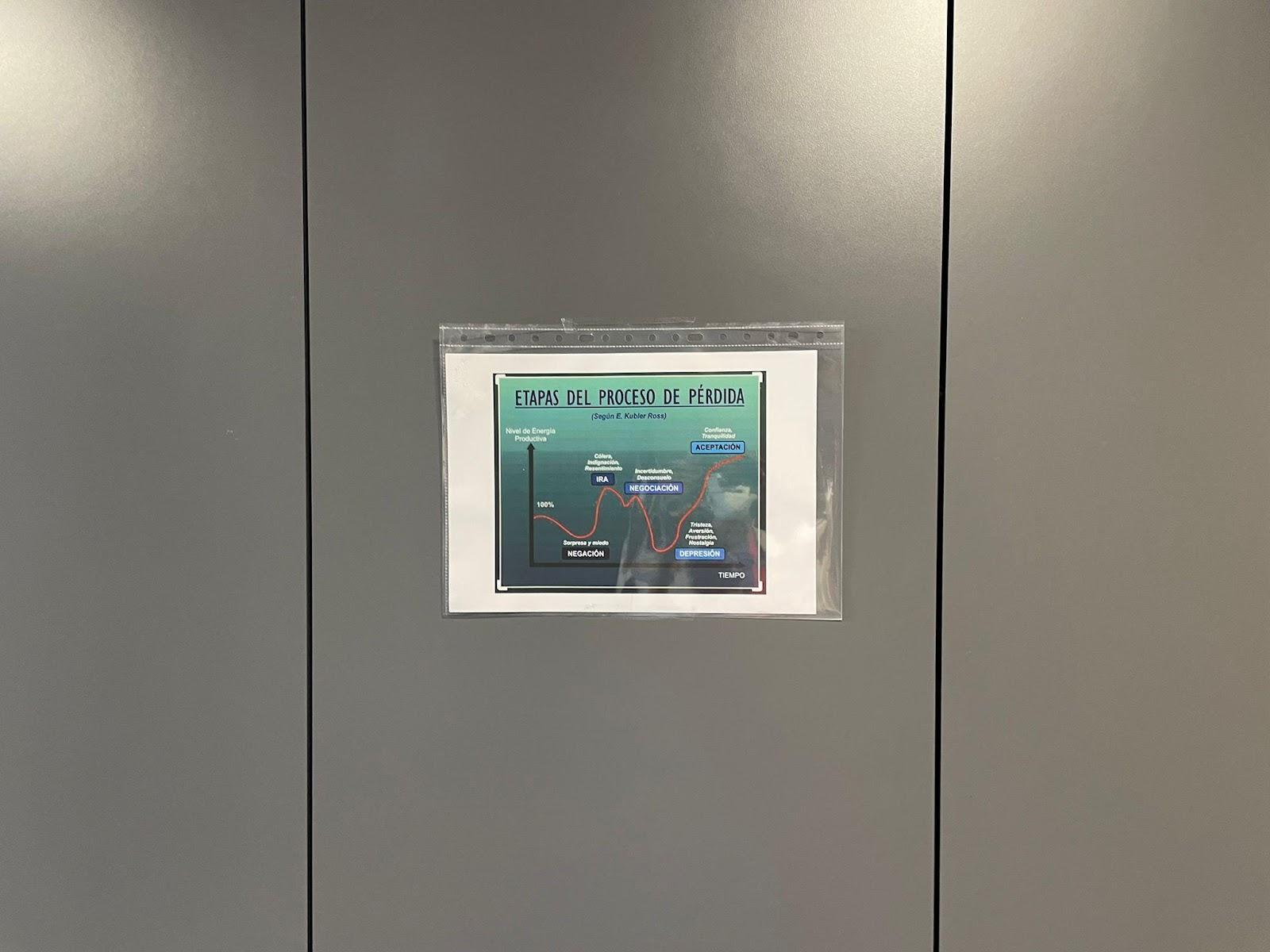
**

Picture 6

Picture 5

(Picture 5 – Hospital waiting room with Ricardo’s messages; Picture 6 – Küebler Ross Loss and Grief scheme).

The **Fourth Room** was the **Palliative Care Unit** (Picture 7). This moment was the player’s first contact with the words‘Palliative Care’. Right at the entrance they found a wheelchair, and one player had to sit in it. The other players had to tie the wheelchair-bound player’s hands and feet to it, to ensure that the players could feel how a person with ELA experiences the fact they cannot move their arms and legs. A video of the palliative care team is shown (video 3). The palliative care team spoke about the disease of Ricardo and family necessities. The palliative team cared for Ricardo and his family for more than two years, and now it was important that all the players can help to take care of him. The player in the wheelchair had to answer four true or false questions on Ricardo’s computer using only his eyes ([Head Mouse](https://www.tecnologiasaccesibles.com/en/content/headmouse) program, a free-of-charge virtual mouse designed specifically for people with mobility problems). Correct answers allowed the other players to free their partner from the wheelchair. To do so, however, they needed to open Ricardo’s safe box, which was locked. To find its code, players read Ricardo’s medical report, which had a clinical number. This number opened a cryptex, and inside was a paper with the safe box code. Inside the safe box were paintings, a photo of Maria, a photo of Ricardo’s parents, a cuddly toy, a notebook, a sheet with a painting, and scissors to cut off the ties. Players were invited to decorate Richard’s Room with his belongings and vital memories to fill his life with life. A photo of Ricardo appeared, and they all had to take a picture together with Ricardo’s life values. If players agreed, they could post it on Ricardo’s Instagram writing #LlenaDeVidaTuVida (means: Fill your Life with Life).

**
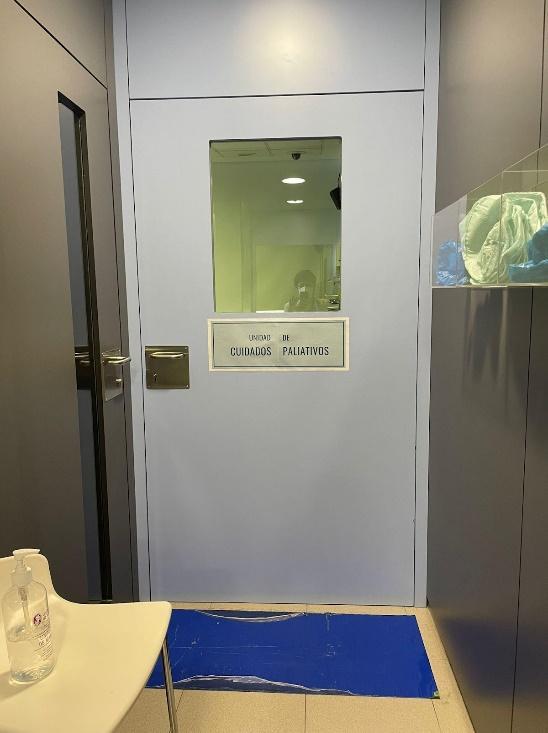
** (Picture 7 – Palliative Care Unit)

Picture 7

In the **Fifth Room (Final Message)**, Maria (Ricardo’s carer and fiancée) thanked the participants for coming (video 4). She explained the benefits of Palliative Care and what this team added to the quality of life of Ricardo and his family. Maria invited the young people to be part of a movement that gives voice to Palliative Care (Picture 8) and, together with her, to be ambassadors for the messages they had received from this experience. This space was full of posters and information about palliative care and had four blank posters with questions and post-its. Participants could write or draw their feelings about the experience, showing how they felt they identified with this story and what they would do to promote the message of Palliative Care in society.

**
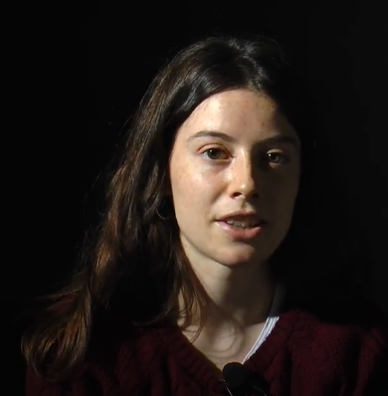
**

Picture 8

(Picture 8– Maria asks young people to give a voice to palliative care)

At the end of the experience, a researcher ran with them a 10-minute debriefing which was recorded for later analysis.

**Step 2. Game ground-rules**

At least one member of the group had to connect to the master games Instagram to be able to access clues and ask questions during the game. The aim of the game was to get to know Ricardo’s life values, and for that they had to stay in the room to decipher the riddles in teamwork. As, due to the theme, we played with words and the concept is a Stay Room, the time factor, which is relevant in escape rooms, was not relevant here. We chose not to include countdowns or stopwatches because the aim is for them to stay and take over Ricardo’s life. By not having to escape from a situation and having to stay, the games were based on motivation and sensation, on finding letters, numbers, clues, observing the objects in the scenarios of the protagonist’s life; also, on listening, through the videos shown in each room.

**Step 3. Practical Issues**

The following materials were created for the Palliative Care Stay Room:

- Four Videos: A student of Pedagogy and Theatre from our University was hired to play the role of Ricardo. With him, two videos of about 2 minutes were recorded: Video 1 – Ricardo’s presentation (projected in Room 1) and Video 2 –Revelation of the diagnosis (projected in Room 2). Video 3 was made with four palliative care professionals (physician, nurse, psychologist, and social worker) who revealed that they accompanied Ricardo and his family and explained what palliative care is (projected in Room 3). Video 4 was made with a nursing student who acted as Maria (Ricardo’s fiancée). She gave her testimony of gratitude to the palliative care professionals and invited students to be ambassadors of this type of care, challenging them to pass a real and positive message to others (projected in Room 5).
- Ricardo’s Instagram Account.
- Activity disclosure posters, online questionnaires (registration and consent, TECA empathy scale, activity assessment).

The following key stakeholders were involved:

- Tantaka – A solidarity-time association of university student volunteers to help in the promotion and execution of the Palliative Care Stay Room.
- Social Innovation Unit of Navarra (Spain) – A public-private association related to the regional Government, made up of 18 partners across different disciplines, that supports innovation and entrepreneurship. UISN was an adviser and helped to give visibility to the Palliative Care Stay Room.
